# Circ_0005615 restrains the progression of multiple myeloma through modulating miR-331-3p and IGF1R regulatory cascade

**DOI:** 10.1186/s13018-023-03832-3

**Published:** 2023-05-12

**Authors:** Qinxin Zhang, Hui Duan, Wupeng Yang, Hao Liu, Xiaoyang Tao, Yan Zhang

**Affiliations:** 1 Department of Spinal Surgery, Ordos Central Hospital, Ordos, 017000 Inner Mongolia China; 2 Center for Local Diseases and Chronic Diseases, Dongsheng District Center for Disease Control and Preventio, Ordos, 017000 Inner Mongolia China; 3 Department of Medical Imaging, Ordos Central Hospital, No. 23, Yijinhuoluoxi Street, Dongsheng District, Ordos, 017000 Inner Mongolia China

**Keywords:** Multiple myeloma, circ_0005615, miR-331-3p, IGF1R

## Abstract

**Background:**

Circular RNAs are implicated in modulating the progression of various malignant tumors. However, the function and underlying mechanisms of circ_0005615 in multiple myeloma (MM) remain unclear.

**Methods:**

The expression levels of circ_0005615, miR-331-3p and IGF1R were tested by quantitative real-time polymerase chain reaction or western blot assay. Cell counting kit-8 and 5-ethynyl-2′-deoxyuridine (EdU) assay were performed for cell proliferation detection. Cell apoptosis and cell cycle were measured by flow cytometry. The protein expressions of Bax and Bcl-2 were detected by western blot assay. Glucose consumption, lactate production and ATP/ADP ratios were estimated to disclose cell glycolysis. The interaction relationship among miR-331-3p and circ_0005615 or IGF1R was proved by dual-luciferase reporter assay.

**Results:**

The abundance of circ_0005615 and IGF1R was increased in MM patients and cells, while the expression of miR-331-3p was decreased. Circ_0005615 inhibition retarded the proliferation and cell cycle progression, while reinforced the apoptosis of MM cells. Molecularly, circ_0005615 could sponge miR-331-3p, and the repressive trends of circ_0005615 deficiency on MM progression could be alleviated by anti-miR-331-3p introduction. Additionally, IGF1R was validated to be targeted by miR-331-3p, and IGF1R overexpression mitigated the suppressive function of miR-331-3p on MM development. Furthermore, IGF1R was mediated by circ_0005615/miR-331-3p axis in MM cells.

**Conclusion:**

Circ_0005615 downregulation blocked MM development by targeting miR-331-3p/IGF1R axis.

**Supplementary Information:**

The online version contains supplementary material available at 10.1186/s13018-023-03832-3.

## Introduction

Multiple myeloma (MM) is a hematological malignancy, which originates from human plasma cells in bone marrow and is distinguished by malignant growth of plasma cells [1, 2]. With the elevation of the elderly population in china, the incidence of MM is on the rise [3]. The main treatment strategies for MM patients contain chemotherapy and radiotherapy. Although these treatment method have achieved good results, the survival rate and treatment effect of MM are still unsatisfactory [4]. Therefore, identifying new mechanisms involved in the malignant phenotypes of MM and revealing the molecular regulatory network of MM development are of great significance for investigating more effective diagnosis and treatment strategies for MM patients [5].

Circular RNAs (circRNAs) are novel noncoding RNAs with covalently closed structures [6]. Different from linear RNAs, circRNAs are very stable [7]. A recent research has disclosed that microRNAs (miRNAs) [8], which are competitively sponged by circRNAs, modulate mRNA level and thus mediate cellular biological behavior [9, 10]. Numerous literature have validated that circRNAs were applied in the regulation of the many diseases progression, including MM [11]. For instance, Liu et al. validated that the circ_0000142 level was increased in MM patients, and depletion of circ_0000142 constrained proliferation and invasion of MM cells via miR-610 and AKT3 regulatory cascade [12]. Wang et al. found that overexpression of circ_0007841 aggravated MM cell growth and metastasis by reducing miR-338-3p and elevating the abundance of BRD4 [13]. Chen et al. stated that circ-CDYL was proved to be up-regulated in MM patients, and circ-CDYL deficiency markedly hindered tumor progression by regulating the miR-1180/YAP signaling cascade [14]. Consequently, circRNAs might be ideal candidates for targeted therapeutic in MM. Nevertheless, the functions and mechanisms of multiple aberrantly expressed circRNAs in MM have not yet been revealed and elucidated. Through the analysis of GEO database (GSE133058), we displayed that there are many abnormally expressed circRNAs. Among them, we found that circ_0005615 (hsa_circRNA_101835) was strikingly increased in MM patients. A previous work by Ma et al. demonstrated that up-regulation of circ_0005615-reinforced proliferation and metastasis of cervical cancer cells by mediating the miR-9-5p/SDC2 axis [15]. Circ_0005615 promoted the progression of MM by targeting the miR-185-5p/IRF4 pathway [15]. However, the specific mechanism of circ_0005615 in the regulation of MM tumorigenesis was not fully understood. Additionally, our study also identified that circ_0005615 was up-regulated in MM patients and cells. Therefore, we hypothesized that circ_0005615 might be involved in the regulation of malignant progression of MM. Besides that we will also disclose the underlying mechanism of circ_0005615 via the circRNA/miRNA/mRNA signaling cascade. We are hopeful that our work will provide novel molecular target for the treatment of MM.

Thus, the aim of this context was to illustrate the action of circ_0005615 in MM tumorigenicity. Meanwhile, we also explored the molecular mechanism of circ_0005615 in modulating the development of MM, which may a new therapeutic target for MM.

## Materials and methods

### Clinical samples

Bone marrow samples form MM patients (*N* = 26) and 26 healthy volunteers were collected from Ordos Central Hospital. This research was legitimated by the Ethics Committee of Ordos Central Hospital, and our study acquired the written informed consent of MM participants.

### Cell cultivation and transfection

Three MM cell lines (U266, MM1.S and NCI-H929) and human normal plasma cells (nPCs) were acquired from American Type Culture Collection (ATCC, Manassas, VA, USA). MM cells were seeded in PRMI-1640 medium (Invitrogen, Carlsbad, CA, USA) supplemented with 10% FBS (Invitrogen) with 5% CO_2_ at 37 °C. Small interfering RNA against circ_0005615 (si-circ_0005615), miR-331-3p mimic (miR-331-3p), IGF1R overexpression vector (IGF1R) and their matched controls were harvested from Genechem (Shanghai, China). The oligo sequences are as follows: si-NC (5′-UCAUACGAACGAGAGAGGAA-3′), si-circ_0005615 (5′-ACCCCUAUAUUUCGAUCUUGA-3′), miR-NC(5′-CGAUCGCAUCAGCAUCGAUUGC-3′), miR-331-3p (5′-GCCCCUGGGCCUAUCCUAGAA-3′), anti-miR-NC (5′-CUAACGCAUGCACAGUCGUACG-3′) and anti-miR-331-3p (5′-UUCUAGGAUAGGCCCAGGGGC-3′). MM1.S and NCI-H929 cells were transfected with 30 nM oligo or 600 ng vector. The transfection experiment was carried out via applying Lipofectamine™3000 Kit (Invitrogen).

### Quantitative real-time polymerase chain reaction (qRT-PCR)

Total RNA was collected by utilizing TRIzol reagent (Invitrogen). For RNase R treatment, a part of RNA was treated by RNase R (Epicentre Technologies, Madison, WI, USA) to manifest the stability of circRNA. Subsequently, the complementary DNA was obtained by reverse transcription kit (Vazyme, Nanjing, China). The RNA expression was evaluated through utilizing SYBR Master Mix (Vazyme) and analyzed by applying 2^−ΔΔCt^ method, and the GAPDH, β-actin and U6 were utilized for normalization. The primers are displayed in Table 1.

**Table 1:** The sequences of primers used in qRT-PCR

Name	Primers for PCR (5′–3′)
hsa_circ_0005615	
Forward	CACCCTTTACCTGGAGCAAA
Reverse	TGGTAAGCAAAGTGGTGTGG
IGF1R	
Forward	ATGTCCAGGCCAAAACAGGAT
Reverse	CATTCCCCAGCCTGCTGTTA
miR-331-3p	
Forward	GTATGAGGCCCCTGGGCCTATC
Reverse	CTCAACTGGTGTCGTGGAG
GAPDH	
Forward	GGAGCGAGATCCCTCCAAAAT
Reverse	GGCTGTTGTCATACTTCTCATGG
U6	
Forward	CGCTTCACGAATTTGCGTGTCAT
Reverse	GCTTCGGCAGCACATATACTAAAAT
β-actin	
Forward	TGGATCAGCAAGCAGGAGTA
Reverse	TCGGCCACATTGTGAACTTT

### Cell proliferation assay

Cells were cultivated in 96-well plates after transfection. Afterward, cell counting kit-8 (CCK-8; Solarbio, Beijing, China) solution was applied to incubate cells. Cell viability was examined via the application of microplate reader at 450 nm (Biotek, Winooski, Vermont).

For 5-ethynyl-2′-deoxyuridine (EdU) experiment, EdU Detection Kit (RiboBio, Guangzhou, China) was applied for cell proliferation test basing on the manufacturer’s instructions. EdU-positive cells were captured through utilizing a fluorescence microscope (Leica, Wetzlar, Germany).

### Flow cytometry assay

According to the instructions of apoptosis detection kit (Solarbio), Annexin V-FITC and propidium iodide (PI) was used to incubate MM1.S and NCI-H929 cells. Subsequently, flow cytometer (Thermo Fisher Scientific) was applied for cell apoptosis detection. In the cell cycle detection experiment, cells were stained with PI using cell cycle detection kit (Solarbio), and cell cycle was also estimated by flow cytometry. Flow cytometry tests were independently repeated three times.

### Western blot assay

Western blot was executed as previously described [16]. Shortly, RIPA Lysis Buffer was adopted to extract total proteins. Later on, protein samples were loaded onto 10% SDS-PAGE gel and then transferred to a polyvinylidene fluoride (PVDF) membrane. Subsequently, the membrane was incubated with primary antibodies against Bax (ab243140, 1:500), Bcl-2 (ab32124, 1:1000), IGF1R (ab131476, 1:1000) and β-actin (ab6276, 1:3000) were procured from Abcam (Cambridge, MA, USA). In addition, secondary antibody (ab6702, 1:3000) was used for western blot analysis. Finally, enhanced chemiluminescence kit (Solarbio) was utilized to visualize the protein bands.

### Detection of glycolysis

By the application of Glucose Assay Kit and Lactic Acid Kit (Biovision, Milpitas, CA, USA) according to the supplier’s protocol, relative glucose consumption and lactate production were investigated. ATP/ADP Ratio Analysis Kit (Keygen Biotech, Nanjing, China) was applied to evaluate the relative ATP/ADP ratios.

### Dual-luciferase reporter assay

The targeted relationships among miR-331-3p and circ_0005615 or IGF1R are displayed through the starbase website. The wild-type (WT) and mutant-type (MUT) luciferase plasmids WT-circ_0005615, MUT-circ_0005615, WT-IGF1R 3′UTR and MUT-IGF1R 3′UTR were constructed via cloning the wild-type or mutant sequences into the pmirGLO vector (Promega, Madison, WI, USA). The miR-331-3p or miR-NC and plasmid were co-transfected into MM1.S and NCI-H929 cells. Luciferase activity was gauged by Dual-Lucy Assay Kit (Solarbio).

### Statistical analysis

Statistical analysis was employed via applying Student’s *t* test or the analysis of variance. The correlation among miR-331-3p and circ_0005615 or IGF1R was assessed by Pearson’s correlation analysis. Statistically significant were indicated as *P < 0.05, **P < 0.01, ***P < 0.001 or ****P < 0.0001.

## Results

### Circ_0005615 level was up-regulated in MM samples and cells

**Fig. 1:**
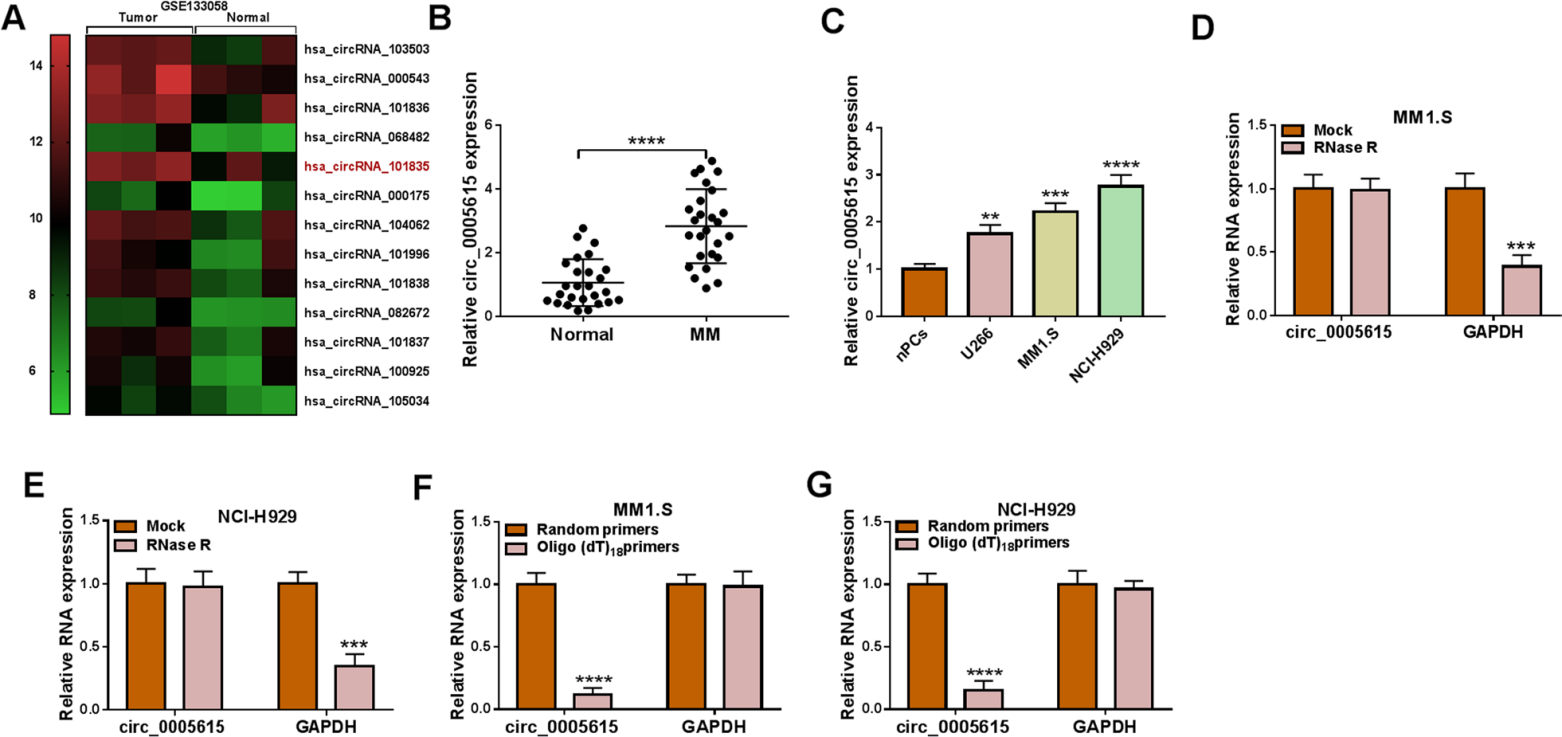
Circ_0005615 was highly expressed in MM samples and cells. **A** Heat maps displaying the 13 up-regulated circRNAs in MM patients (GSE133058). Green showed low expression and red represented high expression. **B** QRT-qPCR was executed to examine the level of circ_0005615 in 26 pairs of MM samples and healthy volunteers. **C** QRT-qPCR analysis of circ_0005615 abundance in MM cells and normal nPCs cells. **D**–**E** After RNase R treatment, qRT-PCR was implemented to reveal the levels of circ_0005615 and linear RNA GAPDH in MM1.S and NCI-H929 cells. **F**, **G** Random and Oligo(dT)18 primers were utilized to measure the level of circ_0005615 and GAPDH in reverse transcription. **P < 0.01, ***P < 0.001, ****P < 0.0001

By GEO database (GSE133058: https://www.ncbi.nlm.nih.gov/geo/geo2r/?acc=GSE133058) analysis, 13 abnormally expressed circRNAs in MM samples were screened out, and we found that the abundance of circ_0005615 (hsa_circRNA_101835) was elevated in MM patients (Fig. 1A, B). Also, the increase of circ_0005615 was revealed in MM cell lines (U266, MM1.S and NCI-H929) compared with nPCs (Fig. 1C). The stability of circ_0005615 was gauged by exposure of RNase R. The findings manifested that circ_0005615 was more resistant to RNase R than linear RNA GAPDH in MM1.S and NCI-H929 cells, confirming that circ_0005615 was stable (Fig. 1D, E). Taken together, circ_0005615 might modulate MM development. In addition, oligo (dT) 18 and random primers were adopted for reverse transcription experiments, and the outcomes uncovered that the level of circ_0005615 was lower when oligo (dT) 18 primers were utilized (Fig. 1F, G).

### Circ_0005615 deficiency retarded the proliferation, cell cycle progression and glycolysis of MM cells and aggravated apoptosis

**Fig. 2:**
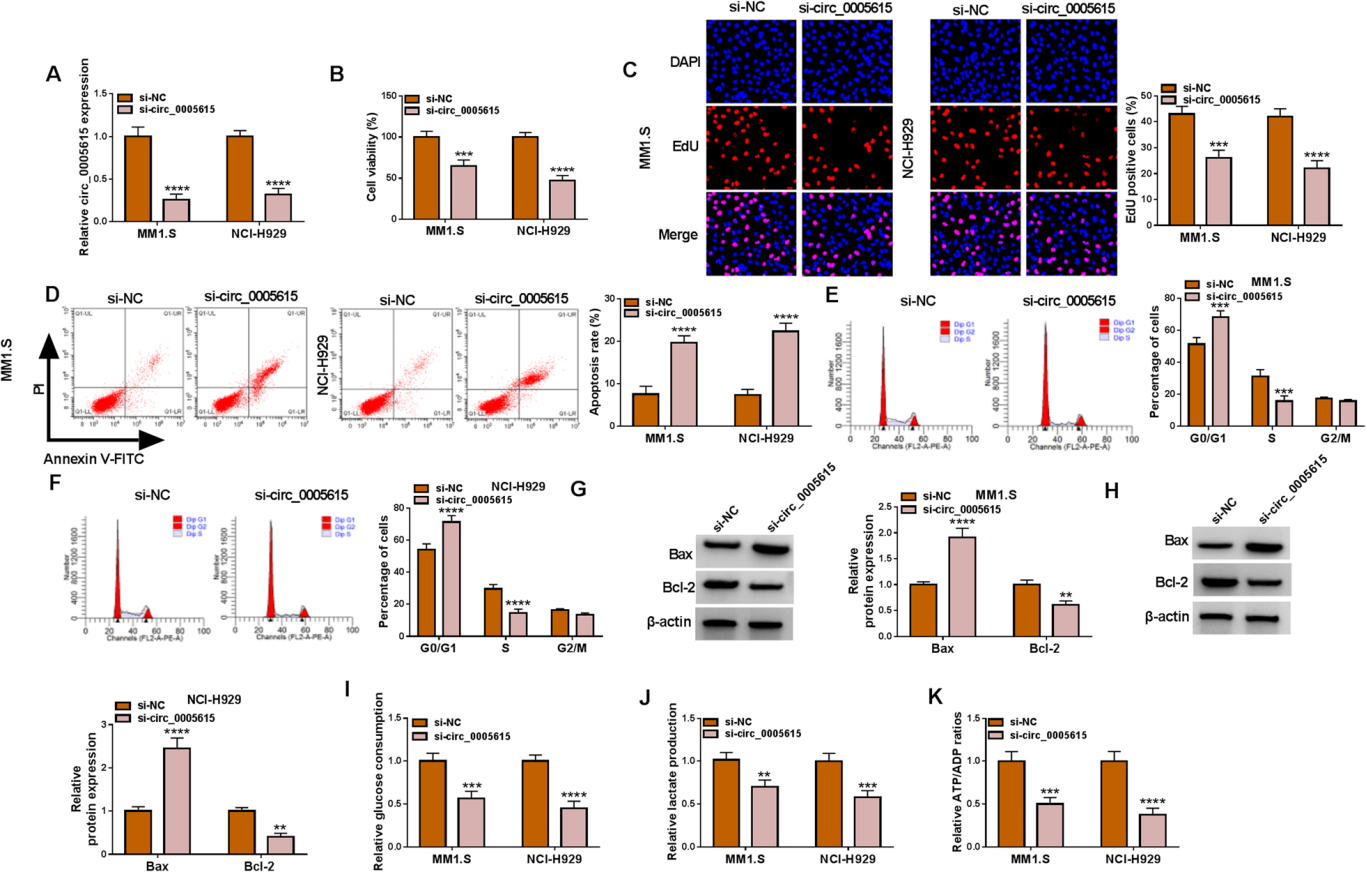
Circ_0005615 depletion curbed cell proliferation, cell cycle progression and glycolysis whereas reinforced apoptosis in MM. MM1.S and NCI-H929 cells were transfected with si-NC and si-circ_0005615, individually. **A** The abundance of circ_0005615 was estimated by using qRT-PCR. **B** Cell viability was evaluated using CCK-8 assay. **C**–**F** Cell proliferation, apoptosis and cell cycle was examined via utilizing EdU assay and flow cytometry. **G**–**H** Western blot assay was employed to explore the protein levels of Bax and Bcl-2. **I**–**K** Cell glycolysis was analyzed by detecting glucose consumption, lactate production and ATP/ADP ratio through the matched kits. **P < 0.01, ***P < 0.001, ****P < 0.0001

To investigate the role of circ_0005615 in MM cells, loss-of-function tests were carried out via depletion of circ_0005615. Contrasted with the si-NC group, circ_0005615 inhibition drastically declined the level of circ_0005615 in MM cells (Fig. 2A). CCK-8 assay and EdU staining identified that circ_0005615 knockdown conspicuously confined the proliferation of MM1.S and NCI-H929 cells (Fig. 2B, C). Subsequently, flow cytometry verified that circ_0005615 silencing notably facilitated cell apoptosis (Fig. 2D), and cell cycle arrested at G0/G1 phase by interference of circ_0005615 (Fig. 2E, F). Additionally, western blot assay was used to estimate the apoptosis-related mark protein, and the data showed that downregulation of circ_0005615 specially increased Bax levels and evidently decreased Bcl-2 expression (Fig. 2G, H). In addition, circ_0005615 deficiency mitigated glycolysis process by declining glucose consumption, lactate production and ATP/ADP ratios (Fig. 2I–K). These outcomes verified that circ_0005615 depletion hampered the tumorigenesis of MM.

### MiR-331-3p was a target of circ_0005615 in MM cells

**Fig. 3:**
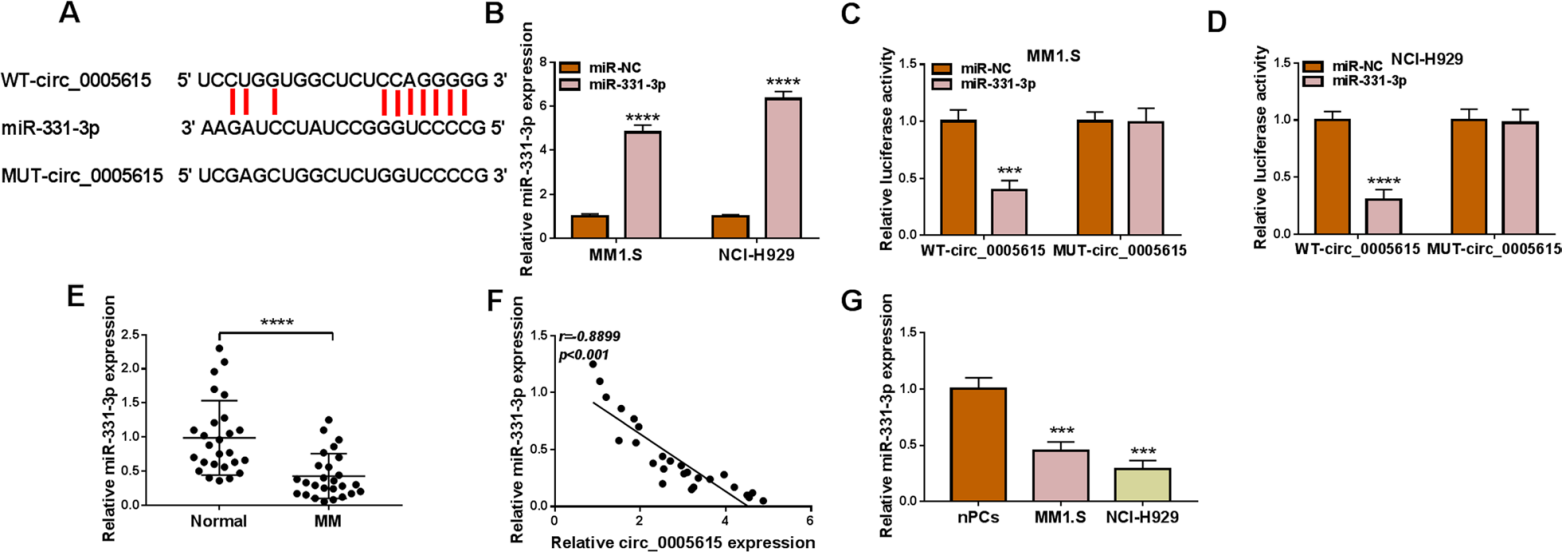
Circ_0005615 sponged miR-331-3p. **A** The binding sites between circ_0005615 and miR-331-3p were exerted. **B** QRT-PCR was conducted to test the transfection efficiency of miR-331-3p mimic. **C**, **D** The interaction between miR-331-3p and circ_0005615 was demonstrated via Dual-luciferase reporter assay. **E** The level of miR-331-3p in MM patients and healthy volunteers was investigated by qRT-PCR. **F** Pearson correlation analysis was exploited to illustrate the correlation between circ_0005615 and miR-331-3p. **G** QRT-PCR was performed to calculate the level of miR-331-3p in MM cells and nPCs cells. ***P < 0.001, ****P < 0.0001

By the prediction of starbase database, miR-331-3p was displayed to contain the complementary sequences of circ_0005615 (Fig. 3A). In order to manifest the interaction among circ_0005615 and miR-331-3p, miR-331-3p mimic was successfully transfected into cell (Fig. 3B). Afterward, dual-luciferase reporter assay suggested that miR-331-3p markedly decreased the luciferase activity of WT-circ_0005615 but not the MUT-circ_0005615 in MM1.S and NCI-H929 cells (Fig. 3C, D), which demonstrating the associative relation between miR-331-3p and circ_0005615. Furthermore, miR-331-3p level was found to be decreased in MM patients (Fig. 3E), which was negatively related with circ_0005615 abundance (Fig. 3F). Also, the abundance of miR-331-3p was lower in MM1.S and NCI-H929 cells in contrast to nPCs (Fig. 3G). Overall, circ_0005615 served as a sponge of miR-331-3p in MM.

### MiR-331-3p inhibition counteracted the regulation of circ_0005615 knockdown on MM progression

**Fig. 4:**
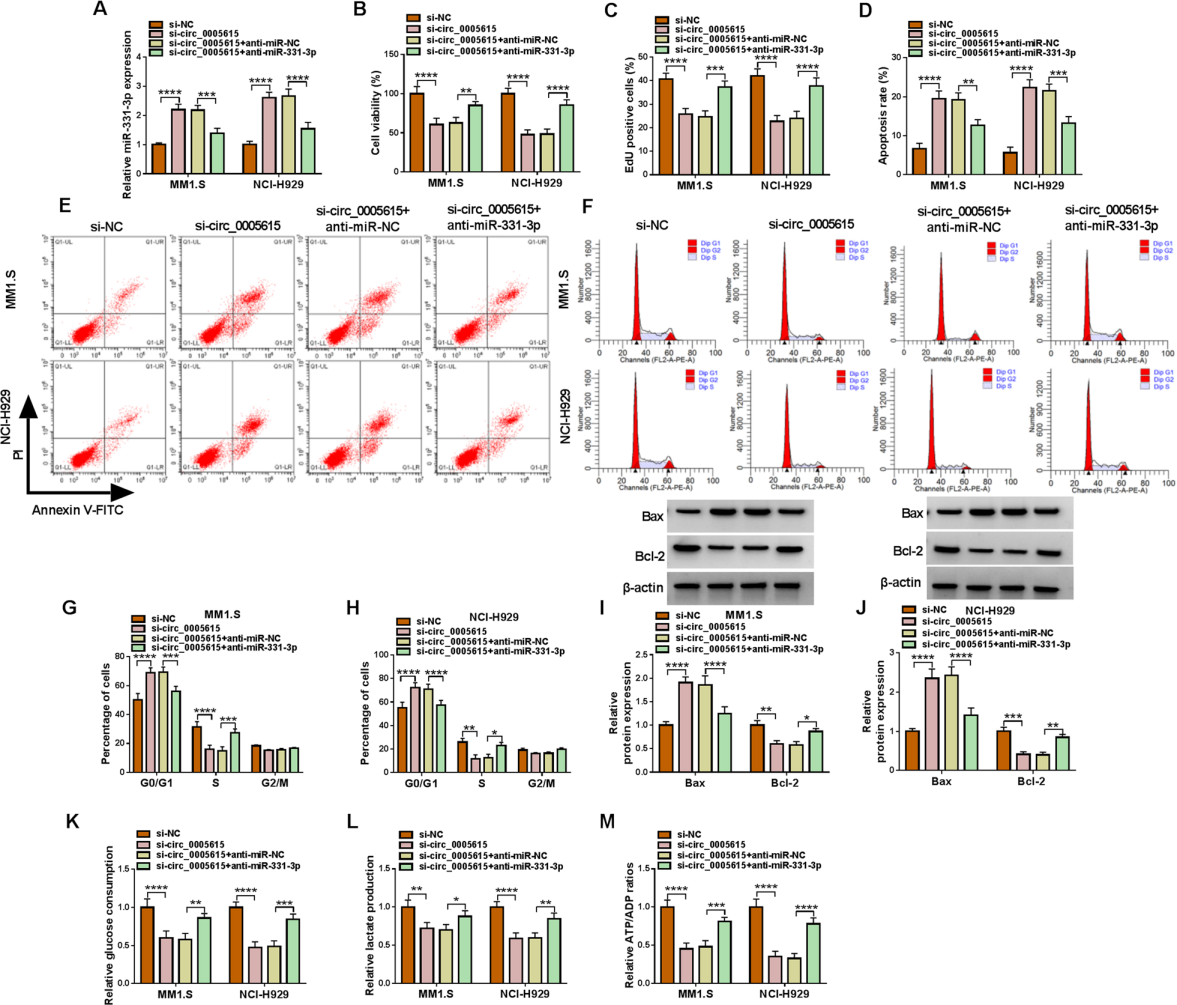
The regulation of circ_0005615 downregulation on MM cell progression could be rescued by miR-331-3p inhibitor. MM1.S and NCI-H929 cells were transfected with si-NC, si-circ_0005615, si-circ_0005615 + anti-miR-NC or si-circ_0005615 + anti-miR-331-3p. **A** QRT-PCR was applied for miR-331-3p expression detection. **B**, **C** CCK-8 assay and EdU staining were exploited to measure cell proliferation. **D**–**F** Flow cytometry was implemented to estimate cell apoptosis and cell cycle. **G**, **H** The protein levels of Bax and Bcl-2 were monitored using western blot analysis. **I**–**K** Glucose consumption, lactate production and the ATP/ADP ratios were calculated to evaluate cell glycolysis. *P < 0.05, **P < 0.01, ***P < 0.001, ****P < 0.0001

To further identify that circ_0005615 mediated MM development by absorbing miR-331-3p, si-circ_0005615 and anti-miR-331-3p were co-transfected into MM1.S and NCI-H929 cells. The results exhibited that the effect of circ_0005615 deficiency on miR-331-3p level was attenuated by anti-miR-331-3p introduction (Fig. 4A). Functional experiments suggested that miR-331-3p silencing could abate the repressive function of circ_0005615 knockdown on MM cell viability and EdU-positive cells (Fig. 4B, C). Additionally, the transfection of anti-miR-331-3p also abolished the elevation of circ_0005615 deficiency on MM cell apoptosis and the inhibitory effect on cell cycle progression (Fig. 4D–H). Circ_0005615 inhibitor also influenced the protein levels of Bax and Bcl-2, which were overturned by of miR-331-3p introduction (Fig. 4I, J). Furthermore, anti-miR-331-3p introduction undermined circ_0005615 downregulation mediated the repression of glycolysis in MM1.S and NCI-H929 cells, as disclosed by measuring glucose consumption, lactate production and the ATP/ADP ratios of MM cells (Fig. 4K, M). Consequently, our results verified that circ_0005615 could sponge miR-331-3p to modulate MM progression.

### IGF1R directly interacted with miR-331-3p

**Fig. 5:**
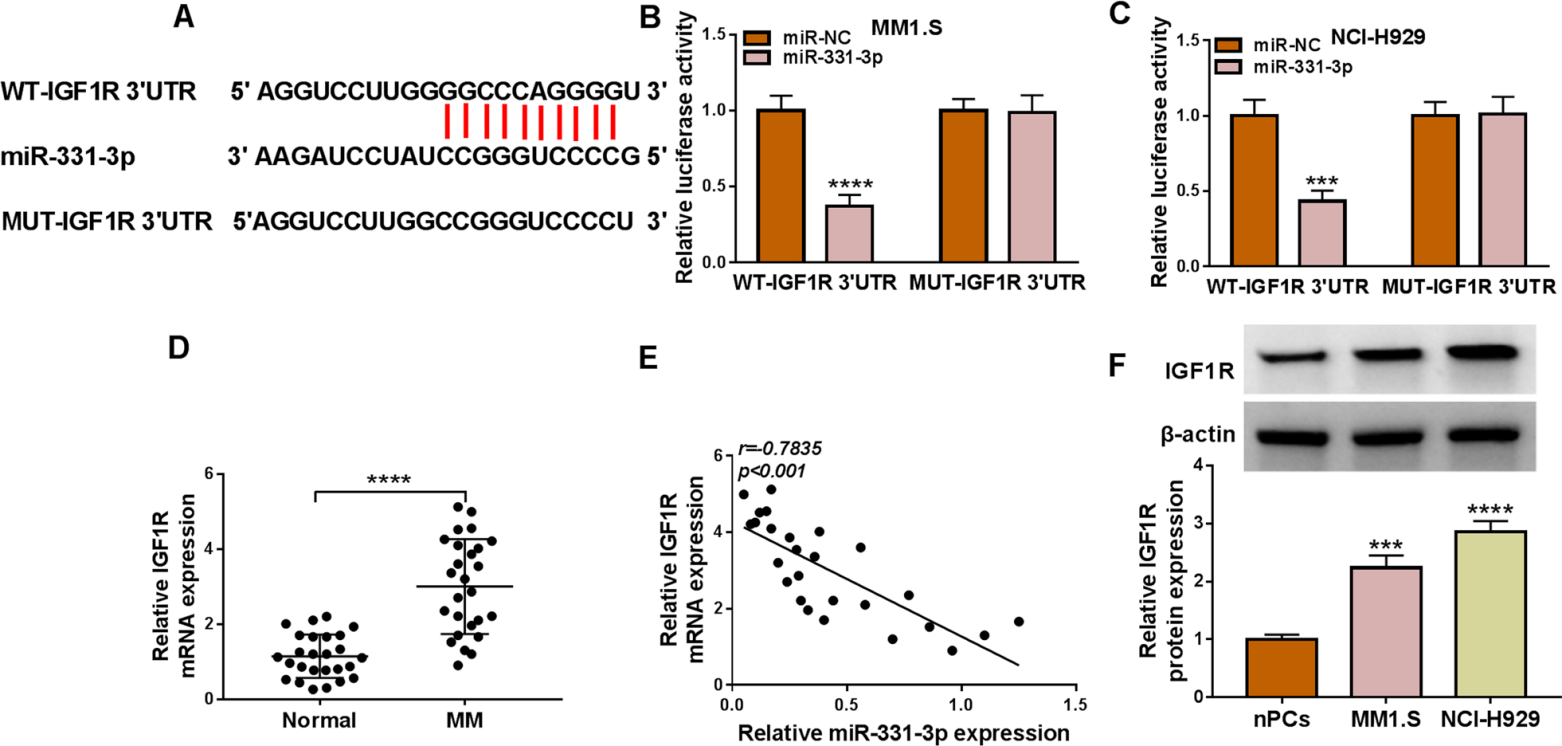
IGF1R was a direct target of miR-331-3p. **A** The binding sites between IGF1R 3′UTR and miR-331-3p were exhibited by starbase. **B**, **C** The binding relationship between miR-331-3p and IGF1R was validated by dual-luciferase reporter assay. **D** IGF1R mRNA level was counted in MM patients and healthy volunteers by qRT-PCR. **E** The correlation between IGF1R and miR-331-3p was determined by Pearson correlation analysis. **F** Western blot was performed for IGF1R protein detection in MM cells. ***P < 0.001, ****P < 0.0001

We also utilized starbase database to explore that IGF1R might be the target of miR-331-3p (Fig. 5A). Simultaneously, the association between IGF1R and miR-331-3p was demonstrated by dual-luciferase reporter assay. The findings showed that IGF1R could bind with miR-331-3p at the predicted wild-type sites (Fig. 5B, C). In addition, IGF1R mRNA level was apparently elevated in MM patients compared with the healthy volunteers by qRT-PCR analysis (Fig. 5D), and a negative relationship was uncovered among the expression of IGF1R and miR-331-3p (Fig. 5E). Similarly, IGF1R mRNA and protein levels in MM1.S and NCI-H929 cells were also substantially higher than that in nPCs cells (Fig. 5F, Fig. S1A in Additional file 1). These data revealed that miR-331-3p directly interacted with IGF1R.

### IGF1R abolished the impact of miR-331-3p on MM cell progression

**Fig. 6:**
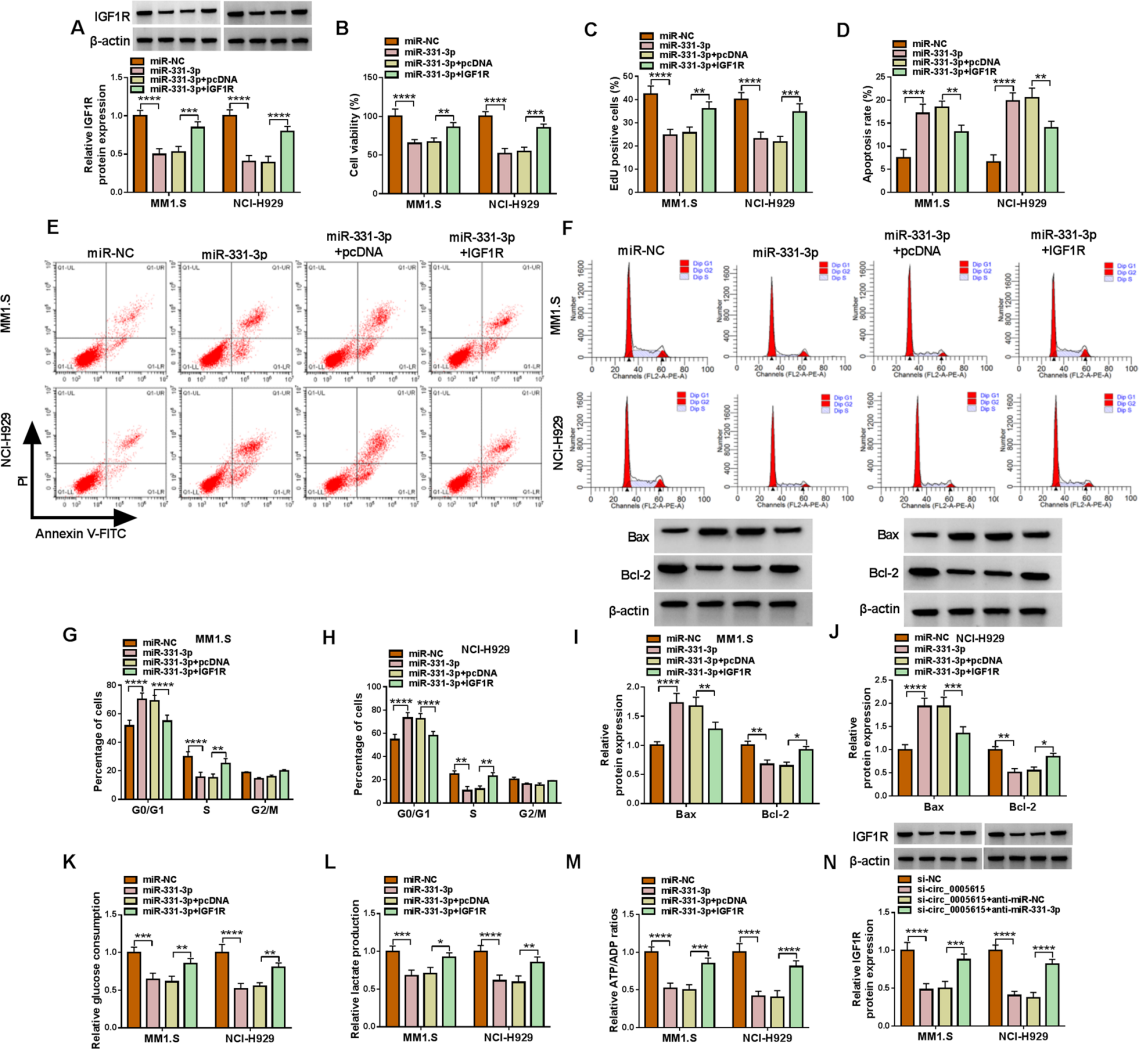
MiR-331-3p overexpression impeded the malignant phenotypes of MM cells partly by reducing the expression of IGF1R. **A**–**L** Cells were transfected with miR-NC, miR-331-3p, miR-331-3p + pcDNA, and miR-331-3p + IGF1R. **A** Western blot assay was carried out for IGF1R level detection. **B**, **C** Cell proliferation was determined by CCK-8 assay and EdU staining. **D**–**F** Cell apoptosis and cell cycle were monitored via flow cytometry. (G-H) The protein expression of Bax and Bcl-2 were detected. **I**–**K** Corresponding test kits were to assess relative glucose consumption, lactate production and ATP/ADP ratios. *P < 0.05, **P < 0.01, ***P < 0.001, ****P < 0.0001

To elucidate whether miR-331-3p targeted IGF1R to regulate MM development, MM1.S and NCI-H929 cells were transfected with miR-NC, miR-331-3p, miR-331-3p + pcDNA, or miR-331-3p + IGF1R. As exhibited in Fig. 6A and Fig. S1B in Additional file 1, co-transfection of miR-331-3p and IGF1R alleviated the decrease of IGF1R level caused by miR-331-3p mimics. CCK-8 and EdU assays suggested that IGF1R overexpression abrogated the suppressive impact of miR-331-3p mimics on MM cell proliferation (Fig. 6B, C). Moreover, the transfection of miR-331-3p triggered apoptosis and impeded cell cycle progression in MM cells, while co-transfection of miR-331-3p and IGF1R partially weakened these effects (Fig. 6D–H). Besides that IGF1R overexpression reversed the increase of Bax levels and the decrease of Bcl-2 expression induced by addition of miR-331-3p (Fig. 6I, J). Meanwhile, up-regulation of IGF1R regained the inhibitory trends of miR-331-3p mimics on glycolysis in MM cells (Fig. 6K, M). Importantly, circ_0005615 silencing decreased the mRNA and protein levels of IGF1R in MM1.S and NCI-H929 cells, while this impact was neutralized by the transfection of anti-miR-331-3p (Fig. 6N, Fig. S1C in Additional file 1). These data demonstrated that circ_0005615 positively regulated IGF1R via sponging miR-331-3p.

## Discussion

MM is a common malignant tumor in the hematological system, the current treatment of MM is not ideal [4]. Therefore, it is pivotal to investigate effective treatment strategies to improve the prognosis of MM patients. Accumulating studies have verified that circRNAs are abnormally expressed in a variety of human malignancies, including MM [17]. Consequently, further study of the association between circRNAs and MM will contribute to clarify the mechanism of MM tumorigenesis. In this study, we found that circ_0005615 was up-regulated in MM by screening the GEO database. Simultaneously, qRT-PCR was carried out to explore the level of circ_0005615, and the results were consistent with GEO database analysis. Functionally, we manifested that circ_0005615 knockdown hindered MM cell growth and glycolysis of MM cells and exacerbated apoptosis. These findings affirmed that circ_0005615 exerted an oncogene function in MM.

The mechanism by which circRNAs can spongy miRNAs and thereby modulate cellular biological processes has been manifested in many studies [8, 18]. Hence, we further evaluated the possible miRNAs targeted by circ_0005615 through starbase database, and the results verified that miR-331-3p may bind to circ_0005615. Meanwhile, we disclosed that circ_0005615 worked as a molecular sponge of miR-331-3p by dual-luciferase reporter assay. Increasingly reports have proved that miR-331-3p could restrain the malignant progression of many tumors. For instance, circ_0004712 deficiency impeded proliferation and metastasis of ovarian cancer cells through the crosstalk with miR-331-3p/FZD4 axis [19]. Du et al*.* demonstrated that interference of circ_0038646 suppressed proliferation and migration of colorectal cancer cells via up-regulating GRIK3 level through absorbing miR-331-3p [20]. In addition, Li et al*.* claimed that depletion of UCA1 curbed malignant progression of MM by up-regulating miR-331-3p abundance and reducing the abundance of IL6R, suggesting that miR-331-3p might function as a tumor suppressor and thus hinder the malignant phenotypes of tumor [21]. Herein, we manifested that miR-331-3p was declined in MM, and miR-331-3p inhibitor alleviated the suppressive impacts of circ_0005615 knockdown on MM progression, hinting that circ_0005615 indeed served as miR-331-3p sponge, thus regulating MM development.

Then, we revealed that IGF1R was targeted by miR-331-3p in MM cells. Xu et al. demonstrated that let-7b-5p could suggest MM progression via decreasing the abundance of IGF1R [22]. Additionally, miR-335 also retarded the malignant progression of MM by targeting IGF1R, affirming the oncogenic function of IGF1R in MM development [23]. Importantly, IGF1R has been shown to be implicated in the regulation of glycolysis in a variety of tumor. Previous studies have identified that tumor cells usually show a high glycolytic rate, and tumor cells rely on the glycolysis pathway to produce energy for rapid growth [24]. Active glycolysis metabolism is a vital biochemical characteristic of malignant tumor cells [25]. Therefore, targeting glycolytic metabolism may be a promising strategy. Hu et al. verified that overexpression of miR-455-5p hindered the growth, metastasis and glycolysis of hepatocellular carcinoma cells by decreasing IGF1R level [26]. In addition, Wang et al. reported that miR-7 confined the growth and glycolysis of gliomas by inhibiting IGF1R [27]. These results indicated that IGF1R was involved in tumor glycolysis. In this test, IGF1R was manifested to be increased in MM. Furthermore, miR-331-3p impeded the malignant phenotypes of MM cells, which were partly reversed by the restored IGF1R expression. Meanwhile, circ_0005615 inhibition-reduced IGF1R expression, and anti-miR-331-3p introduction partly restored the abundance of IGF1R, indicating that circ_0005615 modulated IGF1R level partly via absorbing miR-331-3p.

**Fig. 7:**
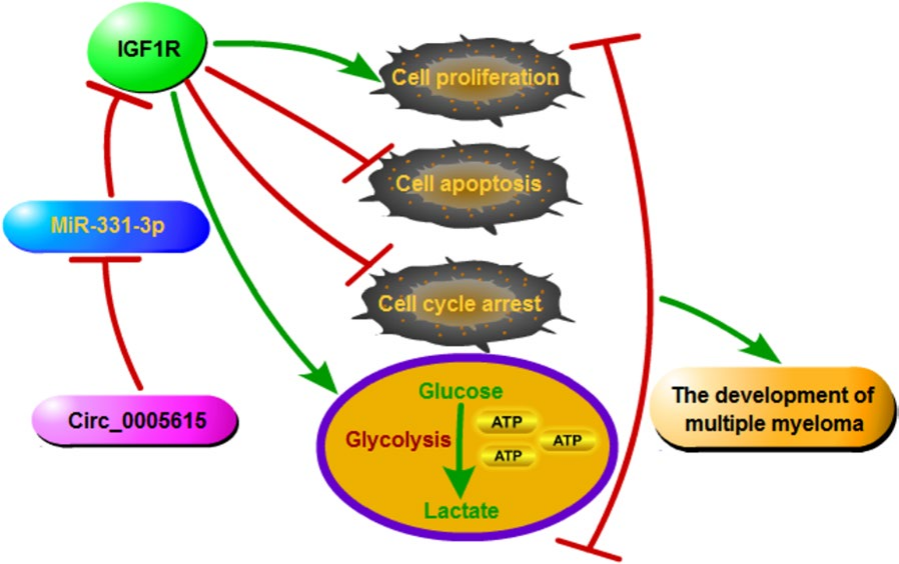
Circ_0005615 promoted MM development by regulating the miR-331-3p/IGF1R axis

Overall, this study disclosed a new circRNA that modulated MM progression. Our work validated that circ_0005615 affected the tumorigenesis of MM by regulating miR-331-3p/IGF1R (Fig. 7), demonstrating that circ_0005615 might be a potential biomarker for MM treatment. Additionally, although the expression of circ_0005615 has been clearly studied using bone marrow samples, exploring the expression of circ_0005615 in bone marrow plasma cells will further clarify the role of circ_0005615 in MM, which was also the limitation of this study.

## Supplementary Information

**Additional file 1: Fig. S1.** New IGF1R mRNA level in MM1.S and NCI-H929 cells was higher than that in nPCs cells, and circ_0005615 silencing decreased the mRNA level of IGF1R in MM1.S and NCI-H929 cells, while this impact was neutralized by the transfection of anti-miR-331-3p. (A-C) QRT-PCR was performed for IGF1R mRNA detection. ***P < 0.001, ****P < 0.0001.

## Data Availability

The datasets used and analyzed during the current study are available from the corresponding author on reasonable request.
