# Supplementary figures and images for: Circ_0005615 restrains the progression of multiple myeloma through modulating miR-331-3p and IGF1R regulatory cascade
Source: J Orthop Surg Res. 2023 May 12;18:356. doi: 10.1186/s13018-023-03832-3 (PMC10176712; doi:10.1186/s13018-023-03832-3)

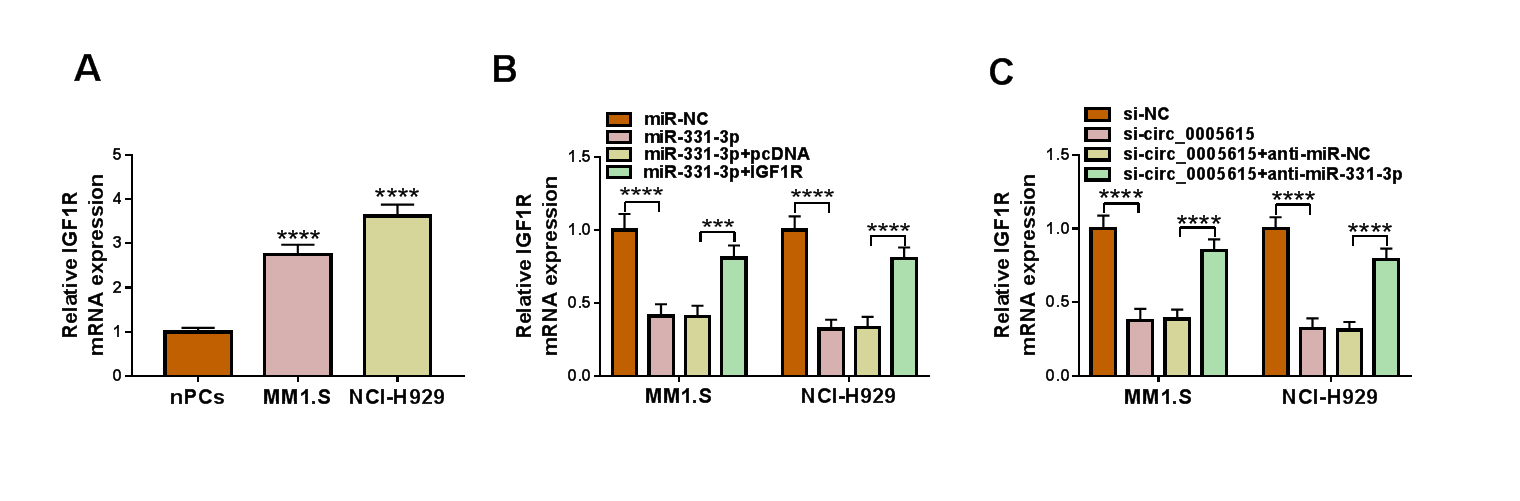

Supplement: Supplementary file 1 — Additional file 1: Fig. S1. New IGF1R mRNA level in MM1.S and NCI-H929 cells was higher than that in nPCs cells, and circ_0005615 silencing decreased the mRNA level of IGF1R in MM1.S and NCI-H929 cells, while this impact was neutralized by the transfection of anti-miR-331-3p. (A-C) QRT-PCR was performed for IGF1R mRNA detection. ***P < 0.001, ****P < 0.0001. [file 13018_2023_3832_MOESM1_ESM.tif]
